# Microfluidic Study of Enhanced Oil Recovery during Flooding with Polyacrylamide Polymer Solutions

**DOI:** 10.3390/mi14061137

**Published:** 2023-05-28

**Authors:** Maxim Pryazhnikov, Andrey Pryazhnikov, Angelica Skorobogatova, Andrey Minakov, Yulia Ivleva

**Affiliations:** 1 Laboratory of Physical and Chemical Technologies for the Development of Hard-to-Recover Hydrocarbon Reserves, Siberian Federal University, 660041 Krasnoyarsk, Russia; apryazhnikov@sfu-kras.ru (A.P.); adskorobogatova@sfu-kras.ru (A.S.);; 2 Laboratory of Heat Exchange Control in Phase and Chemical Transformations, Kutateladze Institute of Thermophysics, 630090 Novosibirsk, Russia

**Keywords:** enhanced oil recovery, polymer flooding, polyacrylamide solution, microfluidics, microfluidic chip, rheology, wettability

## Abstract

A series of experiments have been carried out on the flooding of microfluidic chips simulating a homogeneous porous structure with various displacement fluids. Water and polyacrylamide polymer solutions were used as displacement fluids. Three different polyacrylamides with different properties are considered. The results of a microfluidic study of polymer flooding showed that the displacement efficiency increases significantly with increasing polymer concentration. Thus, when using a 0.1% polymer solution of polyacrylamide grade 2540, a 23% increase in the oil displacement efficiency was obtained compared to water. The study of the effect of various polymers on the efficiency of oil displacement showed that the maximum efficiency of oil displacement, other things being equal, can be achieved using polyacrylamide grade 2540, which has the highest charge density among those considered. Thus, when using polymer 2515 with a charge density of 10%, the oil displacement efficiency increased by 12.5% compared to water, while when using polymer 2540 with a charge density of 30%, the oil displacement efficiency increased by 23.6%.

## 1. Introduction

Polymer flooding constitutes one of the physicochemical methods for enhanced oil recovery [1]. The technology of the method is uncomplicated and superior to other chemical technologies due to relatively low risks. The application range of polymer flooding has significantly expanded in recent years; hence there are examples of their real application in the fields [2,3]. Polymer flooding comprises water injection with the addition of a polymer into the reservoir in order to improve sweep efficiency, which is achieved by increasing viscosity. The increased viscosity of water provides better mobility control between the injected water and the hydrocarbons in the reservoir [4].

The most common polymers for increasing viscosity are considered to be xanthan, hydrophobic associative polymers, polyacrylamides, etc. In the 1970s and 1980s, xanthan (a polysaccharide) generated considerable interest due to its salt resistance and resistance to mechanical decomposition [5]. However, neither inaccessibility nor high prices allowed xanthan to become competitive. What is also worth noting is that microbial degradation of biopolymers is a common problem, and there are still few works on controlling it with the help of proper processing.

In the second half of the last century, hydrophobic associative polymers began to be studied [6]. Associative polymers can provide low viscosity at low temperatures (i.e., near injection wells) and higher viscosity outside the thermal front in the formation (closer to the polymer-oil displacement front). The main concerns with the use of hydrophobic associative polymers are related to the inability to percolate through the reservoir rock under a high-pressure gradient when the polymer is in bound form.

To date, the prevailing polymer is polyacrylamide (PAA) which is used for enhanced oil recovery [7]. Its undoubted advantage over other polymers is availability and price. It is worth noting that several large-scale polymer flooding projects have already been implemented. The largest polymer injection was carried out in 1996 at the Daqing field (China) [8]. Polymer injection at the Shengli and Daqing oil fields has increased oil recovery in the range of 6 to 12%. Another example of successful polymer injection in the 1990s was in the Courtenay field (France), where oil production was reported to increase from 5 to 30% in the secondary production mode [9].

Despite the fact that polymer flooding is recognized as an effective method of enhanced oil recovery, the current challenges of this technology are aimed at optimizing costs and reducing risks when implementing in the fields, as well as a deep analysis of the enhanced oil recovery mechanisms. Nowadays, microfluidic technologies are a new tool for a more meticulous and comprehensive study of the waterflooding process [10,11]. Many physical processes and phenomena occurring at the microscale of pores, which cannot be realized in a rock, can be visually observed with the help of microfluidics. This raises new possibilities in understanding reservoir processes.

Early in its development, microfluidics was used exclusively in microbiology, but today its scope has enlarged significantly in areas such as biomedicine [12], genetics [13], oil and gas industry [14], etc. Microfluidics enables studying the processes of multiphase flow in the pore space of rock micromodels in real time and has an enormous speed of the experiment (many times higher than in traditional experiments).

Today, microfluidics is widely used in practical research. In Refs. [15,16], a rectangular compression-expansion microchannel was used to study the flows of polymer solutions through it. The authors [17] considered active and passive microfluidic devices for sorting model organisms (for example, *Caenorhabditis elegans*); The advantages of microfluidic devices over conventional commercial sorting tools are shown. In Ref. [18], an underwater antigravity superoleophobic “pump” created on the basis of microfluidic system is reported. The microfluidic system can continuously deliver oil from water/oil phase to air/oil phase, which can meet the requirements for the large-area oil collection in oil spills.

The microscopic mechanism of the interaction of carbon dioxide and crude oil using a microfluidic experiment at high temperature and high pressure was analyzed [19]. In [19], the main mechanisms of enhanced oil recovery are the miscibility effect, oil expansion, viscosity reduction, as well as improving the properties of crude oil.

The paper [20] presents a systematic study on the mobilization regimes of oil in a porous medium using polymer-coated silicon dioxide nanoparticles. The conclusion about the effectiveness of polymer-coated silica nanoparticles for enhanced oil recovery in three wettability states (water-saturated, intermediate wetted, and oil wetted) was presented.

Investigation of the potential of nanocellulose (cellulose nanocrystals and cellulose nanofibrils oxidized by TEMPO) as a chemical additive in water for enhanced oil recovery [21]. The results of core flooding were confirmed by microfluidic experiments. The nanofluid resulted in better sweep efficiency compared to brine flooding by breaking up large oil pools and mobilizing them.

In this work, the study of the rheology of polymer solutions is considered. Then, the contact angle and interfacial tension measurements are analyzed. Finally, the results of the flooding of microfluidic chips imitating a homogeneous porous structure using various polymeric solutions of polyacrylamide are presented. The study will allow a better understanding of the process of oil displacement from model microfluidic chips using polyacrylamide solutions. The existing method of laboratory research does not meet the modern requirements of the oil industry. Often, they have a high cost and low repeatability, are performed for a long time, and are not always reliable. This problem is solved with the help of microfluidic technology. This technology will make it possible to carry out experiments in reservoir conditions, successfully supplementing, and sometimes even replacing, core studies.

## 2. Materials and Methods

### 2.1. Polymer Solutions

Anionic polymers based on polyacrylamide of different brands were used in the work: Praestol (CJSC Moscow-Stockhausen, Moscow, Moscow oblast, Russia) and Poliflok (NPO Polyflok, Kemerovo oblast, Russia) with different molecular weights (see Table 1). Solutions with mass concentrations of 0.01, 0.5, and 0.1% were prepared.

To prepare polymer solutions, distilled water was heated to 60–65 °C; a weighed portion of the polymer was slowly poured into a glass beaker with a small part of the water being stirred with an OFITE magnetic stirrer (OFITE, Houston, TX, USA). This solution was subsequently diluted to the required volume and was kept on a magnetic stirrer for 30 min. Then, it was placed on a HAMILTON BEACH (OFITE, Houston, TX, USA) high-speed stirrer for more intensive mixing in order to prevent the formation of polymer clots in the bulk of the solution. The measurements were carried out at 25 °C.

The viscosity coefficient was measured as a function of the shear rate of the studied solutions using a Brookfield DV2T (Brookfield Engineering, Berwyn, IL, USA) rotational viscometer at a temperature of 25 °C according to the method described in [22,23]. The viscosity at a fixed shear rate was obtained by averaging three independent repetitions.

### 2.2. Crude Oil

**Figure 1:**
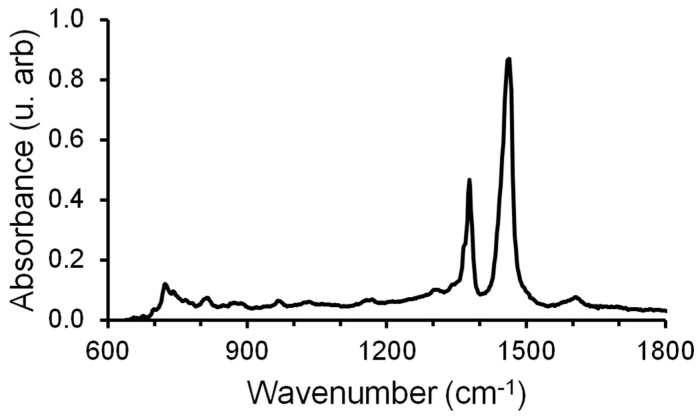
IR spectrum of the crude oil used.

The experiments used an oil sample with a density of 852 kg/m^3^ and a viscosity of 17.8 mPa s. The content of various organic and inorganic substances and their compounds was determined using Fourier transform infrared spectroscopy (FTIR). The FT-IR method is based on the microscopic interaction of infrared light with a chemical substance through an absorption process, and as a result, gives a spectrum unique to a chemical substance, serving as a “molecular imprint” [24]. Figure 1 shows the absorption spectra of various oil samples taken with an IR-Fourier spectrometer Nicolet 6700 (Thermo Fisher Scientific, Waltham, MA, USA). The optical density at the maximum of the absorption bands was determined, as well as the spectral coefficients (see Table 2): C_1_ = D_1600_/D_720_ (aromatics); C_2_ = D_1710_/D_1465_ (oxidation); C_3_ = D_1380_/D_1465_ (branching); C_4_ = (D_720_ + D_1380_)/D_1600_ (aliphatic); and C_5_ = D_1030_/D_1465_ (sulfur content).

The elemental composition of oil was determined by X-ray fluorescence spectrometry Axios-MAX (Malvern Panalytical, Malvern, West Midlands, UK) [25] (see Table 3).

### 2.3. Contact Angle and Interfacial Tension

Interfacial tension σ and contact angle θ were studied using the technique we exploited earlier [26]. The measurements were carried out using an automatic tensiometer IFT-10 (Core Laboratories, Houston, TX, USA) based on the pendant drop method. The interfacial tension σ was determined from the geometric parameters of a hanging oil drop in the test solution. The contact angle θ was measured by the trapped bubble method. The contact angle and interfacial tension were both measured using the DropImage Advanced software (Ramé-hart instrument co., Succasunna, NJ, USA). Interfacial tension and contact angle were measured five times. Then, the data were averaged.

### 2.4. Microfluidic Chip and Experimental Procedure

**Figure 2:**
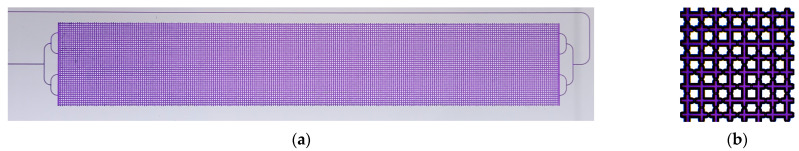
Photographs of a microfluidic chip: (**a**) microfluidic chip with porous media; (**b**) an enlarged fragment of a microfluidic chip.

A microfluidic chip (Dolomite, London, Greater London, UK) imitating the porous structure of the rock was used in our work (Figure 2a). The size of the microfluidic chip is 92.5 × 15.0 mm^2^ and 4 mm thick, and the porous area of the chip is 10 × 60 mm^2^. The porous area is formed by repeating (150 times) a 2 × 2 mm^2^ square (Figure 2b). The arrangement of channels in a square is a grid of 8 × 8 channels, which have an almost elliptical cross-section (channel depth and width of 100 μm and 110 μm, respectively). The channels in the grid have constrictions that are randomly distributed to simulate the natural structure of the rock. The grid contains 38 pores with Ø63 µm, 40 pores with Ø85 µm, and 50 straight channels. The microfluidic device with the characteristic dimensions of the porous structure presented was chosen to model the complex structure of porous sandstone rocks. It is known that sandstone is characterized by relatively high permeability and porosity compared to other types of rock [27].

**Figure 3:**
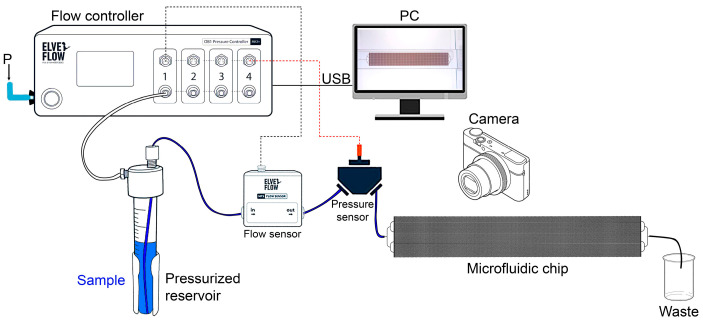
Scheme of the experimental setup.

A detailed description of the experiment is presented in [28]. The installation scheme is shown in Figure 3. The displacement fluid flow was controlled using a multichannel high-performance microfluidic pressure controller (Elveflow, Paris, Île-de-France, France). The controller is equipped with two pressure channels up to 8 bar. Pressure maintenance accuracy is 100 Pa, and response and pressure setting time is 35 ms. The controller requires an external pressure source (compressor) to operate. Compressed air from the pressure controller enters a sealed reservoir with the displacement fluid under test. The microfluidic chip is connected to the reservoir with a 1/16″ OD PTFE tube and placed horizontally on a glass table. A light source is installed below the table, whereas a high-speed camera is installed above.

A fluid flow sensor MFS (Elveflow, Paris, Île-de-France, France) was used, operating in the range from 0.03 to 1000 µL/min with an accuracy of ±5% of the measured value, the sensor response time was up to 70 ms. A pressure sensor MPS (Elveflow, Paris, Île-de-France, France) was also used, measuring in the range from −15 to 100 psi, with an accuracy of ±0.2% of the maximum value.

The following is a description of the procedure for conducting a microfluidic experiment. The empty chip was first completely filled with oil, and then the displacement fluid flooding process took place at a fixed flow rate. Several pore volumes were pumped. The picture of the flooding process was recorded by a high-speed camera. After each experiment, the microfluidic chip was successively thoroughly washed with dichloroethane, isopropanol, distilled water, and purged with air.

## 3. Results and Discussion

### 3.1. Rheology of Polymer Solutions

**Figure 4:**
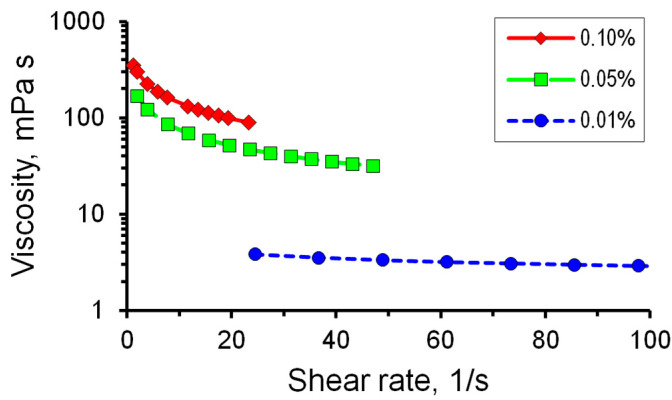
Dependence of the dynamic viscosity of the polymer solution on the shear rate for different concentrations of the A2020 polymer.

Adding polymers to water increases the viscosity of the solution. It is known that polymer solutions of polyacrylamide exhibit non-Newtonian properties. Polymer solutions of polyacrylamides are shear-thinning fluids [29]. This means that the viscosity of the solution depends on the shear rate. Therefore, the dependence of the viscosity of PAA polymer solutions on the shear rate was determined. Figure 4 represents the results of determining such dependencies for solutions with different concentrations of polymer A2020. Figure 4 shows that the addition of PAA to water led to an increase in the viscosity of the solution, and it increased significantly with increasing concentration.

The change in the shear rate has a greater effect on the viscosity values with an increase in the concentration of the polymer in the solution; the viscosity of the solutions decreases with an increase in the shear rate. As for a PAA concentration of 0.01%, the dependence of viscosity on the shear rate is less pronounced compared to concentrations of 0.05 and 0.1%.

The rheology of polymer solutions has a pseudo-plastic behavior. Approximation of the curves of viscosity versus shear rate by the least squares method shows that the rheology of the considered polymer solutions is best described by the two parametric model proposed by Ostwald [30]:

*µ*(*γ*) = *k_v_*(*γ^n^*^−1^)

where *µ* is viscosity, *γ* is shear rate, *k_v_* is consistency index, *n* is flow behavior index. Calculations of rheological parameters (approximation of the viscosity curve) were carried out using the MathCad software (PTC Inc., Boston, MA, USA). The coefficient of determination *R*^2^ was higher than 0.999.

**Figure 5:**
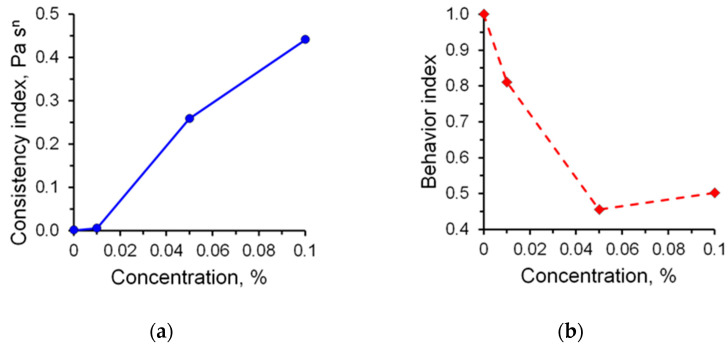
Dependence of the rheological parameters of solutions on the mass concentration of polymer A2020: (**a**) consistency index; (**b**) behavior index.

Studies of the rheological characteristics of A2020 polymer solutions have been carried out. The following regularities were revealed when studying the rheological parameters of solutions. An increase in the proportion of the polymer in the solution significantly affects the rheological characteristics, which are shown in Figure 5. The consistency index increases with an increase in polymer concentration, while the exponent of the power model, on the contrary, decreases.

**Figure 6:**
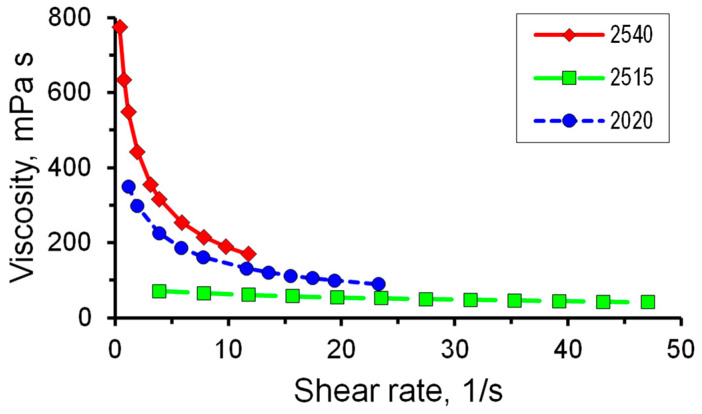
Dependence of dynamic viscosity of 0.1% polyacrylamide solutions on shear rate.

Similar measurements were conducted for polymer solutions containing polyacrylamide of different brands. The mass concentration was 0.1%. The value of the viscosity of solutions with a concentration of 0.1% decreases for samples of all polymers with an increase in the shear rate (see Figure 6). This dependence is most pronounced for PAA 2540; the viscosity changes slightly with varying shear rates for PAA 2515.

**Figure 7:**
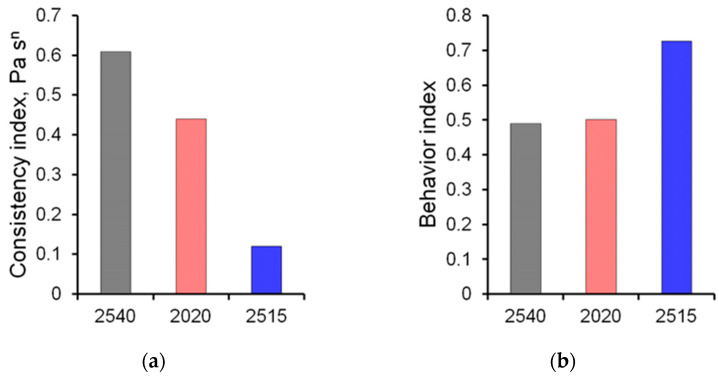
The rheological parameters of 0.1% polymer solutions polymer A2020: (**a**) consistency index; (**b**) behavior index.

A study of the rheological characteristics of 0.1% solutions of polymers of various brands showed that the highest value of the consistency index is observed in the solution of PAA 2540, and the exponent of the power model for this brand of PAA is the lowest. In addition, the lowest value of the viscosity of a 0.1% solution is observed for PAA 2515, while the exponent is higher than for other PAAs. A comparison of rheological parameters is presented in Figure 7.

### 3.2. Wettability Characteristics

Wettability is of great interest for various scientific and industrial applications such as oil extraction, printing, painting, or coating [31]. Wettability describes the balance of interfacial interactions for a solid/liquid system. From a thermodynamic point of view, such a balance can be expressed by the Young equation [32]:

$$\cos\theta =\frac{\gamma_{{sf}_{2}}-\gamma_{{sf}_{1}}}{\gamma_{f_{1,2}}}$$

where *θ* is the equilibrium contact angle, *γ* are interfacial (or surface) tensions, *s* refers to the solid phase, and *f*_1_ and *f*_2_ refer to the two fluid phases.

The solid/fluid surface tension ($\gamma_{{sf}_{2}}$ and $\gamma_{{sf}_{1}}$) cannot be measured directly. Therefore, the one measure of the wettability of a particular liquid is the contact angle. The measurement of the contact angle of a drop on the surface is used as a rapid assessment of the wettability characteristics of the rock. In this case, the angle is estimated between the tangent to the solid–liquid and liquid–liquid interfaces on the three-phase contact line. Hydrophilic (water-attracting) systems are systems in which the contact angle is less than 90°; in hydrophobic (water-repelling), on the contrary, the contact angle is more than 90°.

The effect of polymers on the interfacial tension at the interface “oil-displacing liquid” and the contact angle of the system “displacing liquid-oil-surface” is studied. Slides were used as samples.

**Figure 8:**
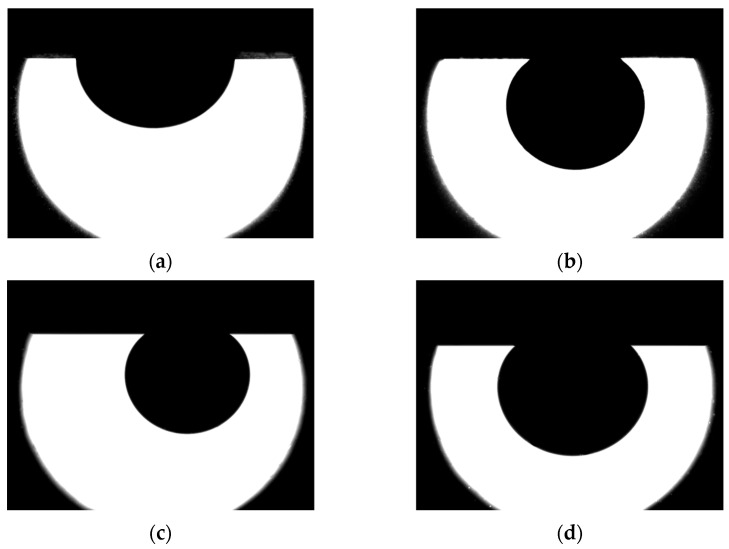
Photographs of an oil drop on rock: (**a**) in water; (**b**) 0.1% solution of polymer A2020; (**c**) 0.1% solution of polymer 2515; (**d**) 0.1% solution of polymer 2540.

The determination of the wetting angle in the system polymer solution-oil-rock was carried out. Figure 8 shows typical photographs for determining the contact angle. The contact angle in the water-oil-surface system was found to be 106° (see Figure 8a). As for polymer solutions, there was an increase in the contact angle, which constituted 161° when using a 0.1% solution of polyacrylamide 2540. As for all considered 0.1% polymer solutions, close values of the contact angle were obtained.

**Figure 9:**
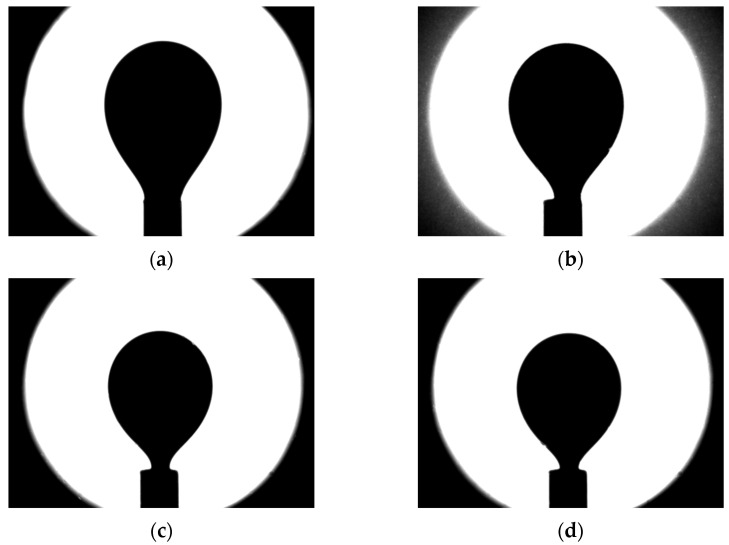
Photographs of an oil pendant drop: (**a**) in water; (**b**) 0.1% solution of polymer A2020; (**c**) 0.1% solution of polymer 2515; and (**d**) 0.1% solution of polymer 2540.

The interfacial tension in the oil-polymer system was measured. Figure 9 depicts photographs of an oil drop in various liquids (water and polymer solutions). The study of the wettability characteristics of polymer solutions showed the following. The addition of the polymer produces a minuscule effect on the interfacial tension, which only slightly increased compared to water. The results of determining the wetting angle and interfacial tension are summarized in Table 4.

### 3.3. Microfluidic Oil Displacement by Polymer Solutions

**Figure 10:**
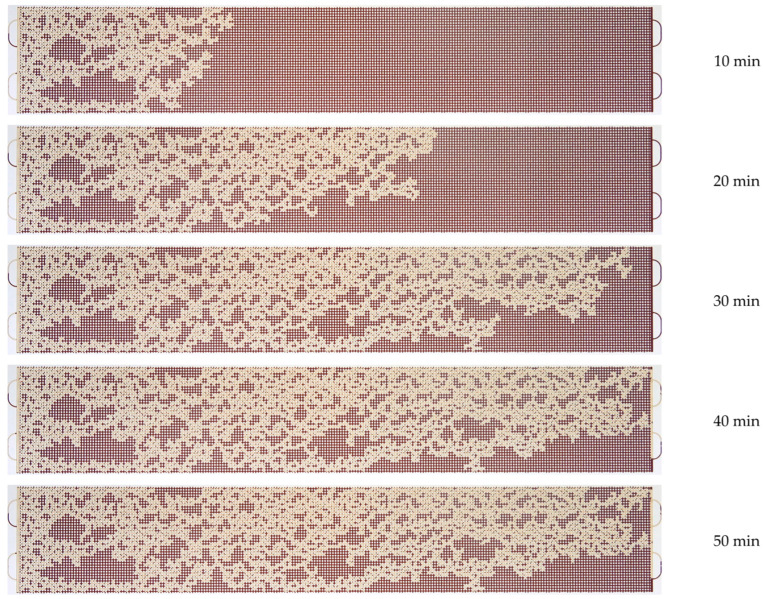
Photographs of the displacement process of an oil sample with a solution of polymer 2540 with a concentration of 0.01%.

**Figure 11:**
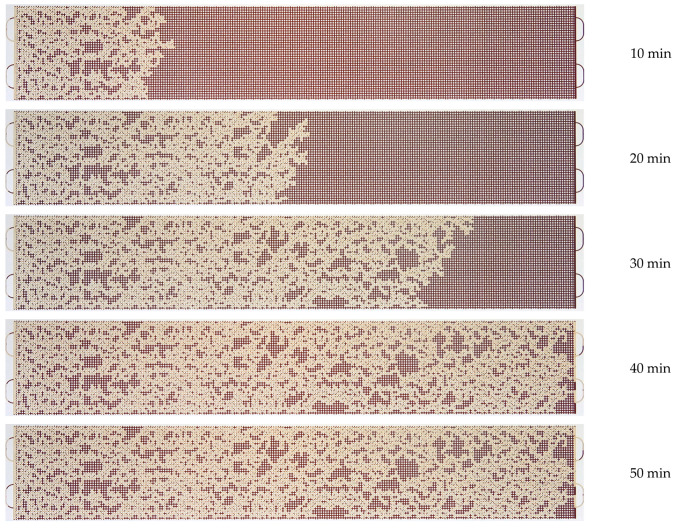
Photographs of the displacement process of an oil sample with a solution of polymer 2540 with a concentration of 0.05%.

**Figure 12:**
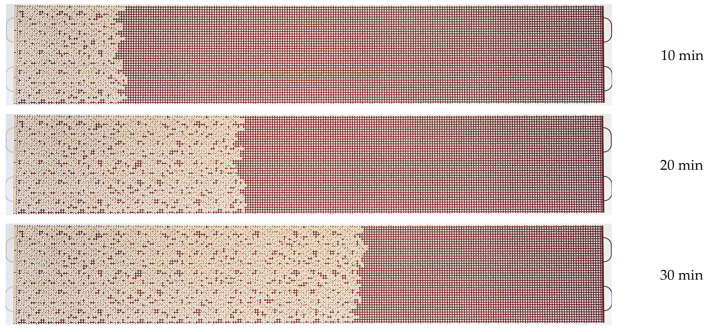

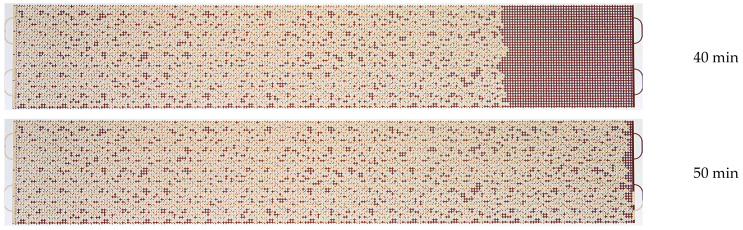
Photographs of the displacement process of an oil sample with a solution of polymer 2540 with a concentration of 0.1%.

A series of microfluidic experiments on oil displacement using polymer solutions has been carried out. An oil sample, as well as several solutions of polyacrylamide polymers, were used as displacement fluids. The solution flow rate was 0.5 µL/min. Figure 10, Figure 11 and Figure 12 show the patterns of oil displacement by solutions with different concentrations of polymer 2540. As can be seen from Figure 10, the front of the solution with a low concentration of polymer 2540 (0.01%) moves uniformly at the beginning of the oil displacement process, and then it begins to change: viscous fingers are formed due to an unstable polymer solution-oil interface. However, the displacement process changes when flooding with solutions with a higher concentration of polymer 2540 (see, for example, Figure 11 and Figure 12). The polymer solution displacement front is broad and uniform.

**Figure 13:**
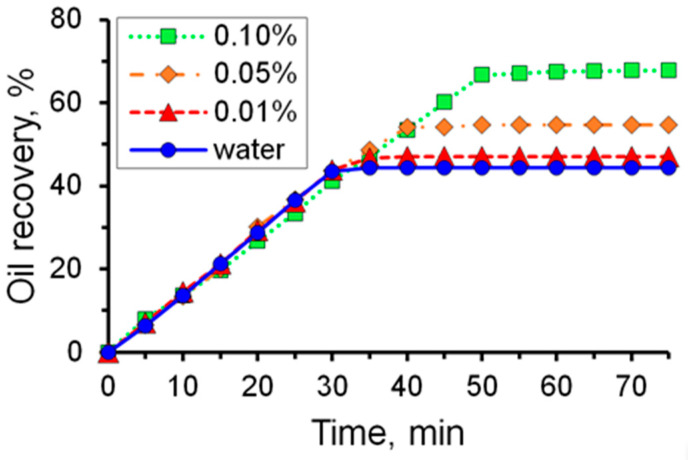
Dependence of the oil displacement efficiency on time when using a solution with different mass concentrations of polymer 2540.

As shown in Figure 10, a 0.01% solution of 2540 breaks through at the exit of the micromodel approximately by the 35th minute. Until this time, the oil displacement efficiency increases almost linearly in proportion to the flow rate of the solution. After the breakthrough of the solution, the flow is established, and the oil displacement coefficient remains practically unchanged in the future. The coefficient of oil displacement from the microfluidic chip with 0.01% solution 2540 was 47%, while in the case of displacement with water, it was 44% (Figure 13). Once the oil displacement process is established (see the last photographs of Figure 10), there are large areas filled with oil.

Further, similar experiments were carried out on flooding a microfluidic chip with solutions of polyacrylamide of different brands (2020 and 2515), differing in molecular weight and charge density. Figures S1 and S2 (see Supplementary Files) explicitly show the dynamics of the process of oil displacement by different polymers at a concentration of 0.1%. A qualitative comparison of the residual distribution of oil and solutions at different concentrations of polymers is presented in Supplementary Files (Figures S3–S5).

**Figure 14:**
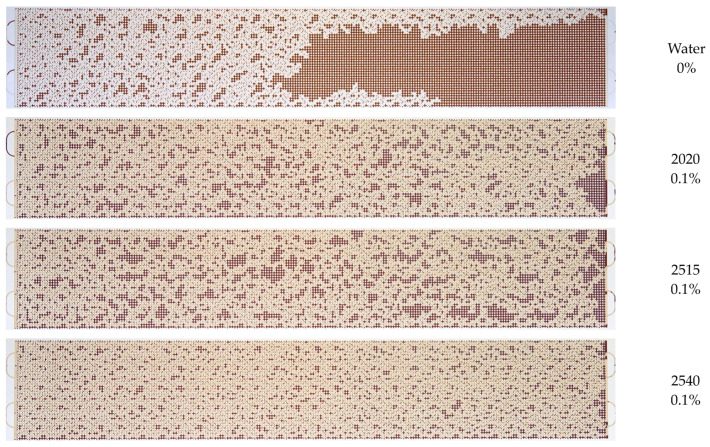
Photographs of the remaining oil distribution in the microfluidic chip after injection of various 0.1% polymer solutions.

**Figure 15:**
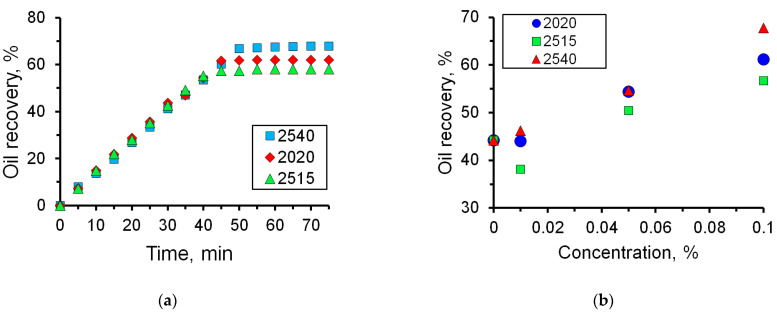
Oil recovery: (**a**) The dependence of the oil recovery on time for 0.1% polymer solutions; (**b**) Oil displacement efficiency as a function of polymer concentration.

It has been established that there is a correlation between the charge density of polyacrylamide and the efficiency of oil displacement (Figure 14 and Figure 15). Thus, when using polymer 2515 with a charge density of 10%, the oil displacement efficiency increased by 12.5% compared to water, while the oil displacement efficiency increased by 23.6% when using polymer 2540 with a charge density of 30%.

## 4. Conclusions

A study of polyacrylamide polymer solutions has been carried out. The rheology of polymer solutions has been measured. As a result, polyacrylamide solutions are pseudoplastic; their rheology is described by a power law. As for the wettability characteristics of polymer solutions, the addition of the polymer does not have a prominent effect on the interfacial tension. The interfacial tension at the interface between polymer solutions and oil slightly increased compared to water. At the same time, the polymer addition has a more significant effect on the wetting angle.

A series of experiments on flooding microfluidic chips imitating a homogeneous porous structure with water and polyacrylamide polymer solutions has been carried out. Additions of water-soluble polyacrylamides increase the viscosity of water injected into porous media. This, in turn, reduces the mobility ratio between oil and water and, thereby, increases the oil displacement efficiency. The results of the microfluidic study showed that the displacement efficiency increases significantly with increasing polymer concentration. Thus, a 23% increase in the oil displacement efficiency was obtained compared to water when using a 0.1% solution of polymer 2540. The study of the effect of various polymers on the oil displacement efficiency showed that the maximum efficiency of oil displacement, other things being equal, can be achieved using a 2540 polymer, which has the highest charge density of polyacrylamide among the considered ones.

Microfluidic studies can significantly speed up laboratory tests, reduce their cost and increase their accuracy. Our current work was aimed at studying the behavior of two-phase flow (polymer solution-oil) in a porous microfluidic chip imitating sandstone during flooding. The advantage of polymer solutions is their rheology. However, there are other flooding fluids available. In further work, we will study the flows of various water-based nanofluids with silicon oxide and aluminum oxide nanoparticles. The goal is to see the effect of nanoparticle size and morphology on displacement efficiency. It is hoped that our future experimental results will provide useful data for comparing traditional displacement fluids (polymer, surfactant, etc.) with nanofluids.

## Figures and Tables

**Table 1:** Polymer characteristics.

Trademark	Molecular Weight, Million Units	Charge Density, %	Hydrolisys Degree, %	Bulk Density, kg/m^3^	Manufacturer
Poliflok A2020	17–20	20	20	600–800	NPO Polyflok
Praestol 2515	8–10	10	-	550–750	CJSC Moscow-Stockhausen
Praestol 2540	12–14	30	-	600–750	CJSC Moscow-Stockhausen

**Table 2:** Optical density and spectral coefficients.

Optical Density *D* at Maximum Absorption Length *λ*, cm^−1^						Spectral Coefficients				
720	1030	1380	1465	1600	1710	C_1_	C_2_	C_3_	C_4_	C_5_
0.119	0.048	0.445	0.846	0.05	0.011	0.420	0.013	0.053	11.30	0.057

**Table 3:** The elemental composition of oil by XRF.

Chemical Element	Intensity, imp/s
Ca	132
S	2484

**Table 4:** Contact angle and interfacial tension.

Displacement Fluid	Polymer Concentration, wt%	θ, deg	$\gamma_{f_{1,2}}$, mH/m
brine	-	105.7	29.9
polymer solution 2515	0.1	154.8	31.6
polymer solution 2540	0.1	161.1	30.4
polymer solution A2020	0.05	159.0	30.1
polymer solution A2020	0.1	158.6	30.6

## Data Availability

Data are contained within the article.

## Supplementary Materials

The following supporting information can be downloaded at: https://www.mdpi.com/article/10.3390/mi14061137/s1, Figure S1: Photographs of the displacement process of an oil sample with a solution of polymer A2020 with a concentration of 0.1%; Figure S2: Photographs of the process of displacing an oil sample with a polymer 2515 solution with a concentration of 0.1%; Figure S3: Photographs of the remaining oil distribution in the microfluidic chip after injection of a solution with different concentrations of polymer 2540; Figure S4: Photographs of the remaining oil distribution in the microfluidic chip after injection of a solution with different concentrations of polymer A2020; Figure S5: Photographs of the remaining oil distribution in the microfluidic chip after injection of a solution with different concentrations of polymer 2515.
