# Supplementary material for: Microfluidic Study of Enhanced Oil Recovery during Flooding with Polyacrylamide Polymer Solutions
Source: Micromachines (Basel). 2023 May 28;14(6):1137. doi: 10.3390/mi14061137 (PMC10301156; doi:10.3390/mi14061137)
Supplement: Supplementary file 1 [file micromachines-14-01137-s001.zip › micromachines-2399737-supplementary.pdf]

Supplementary Material

Microfluidic Study of Enhanced Oil Recovery during Flooding with Polyacrylamide Polymer Solutions

Maxim Pryazhnikov [1,2,*], Andrey Pryazhnikov [1], Angelica Skorobogatova [1], Andrey Minakov [1,2,*] and Yulia Ivleva [1]

[1] Laboratory of Physical and Chemical Technologies for the Development of Hard-to-Recover Hydrocarbon Reserves, Siberian Federal University, 660041 Krasnoyarsk, Russia; apryazhnikov@sfu-kras.ru (A.P.); adskorobogatova@sfu-kras.ru (A.S.)
[2] Laboratory of Heat Exchange Control in Phase and Chemical Transformations, Kutateladze Institute of Thermophysics, 630090 Novosibirsk, Russia
* Correspondence: mpryazhnikov@sfu-kras.ru (M.P.); aminakov@sfu-kras.ru (A.M.)

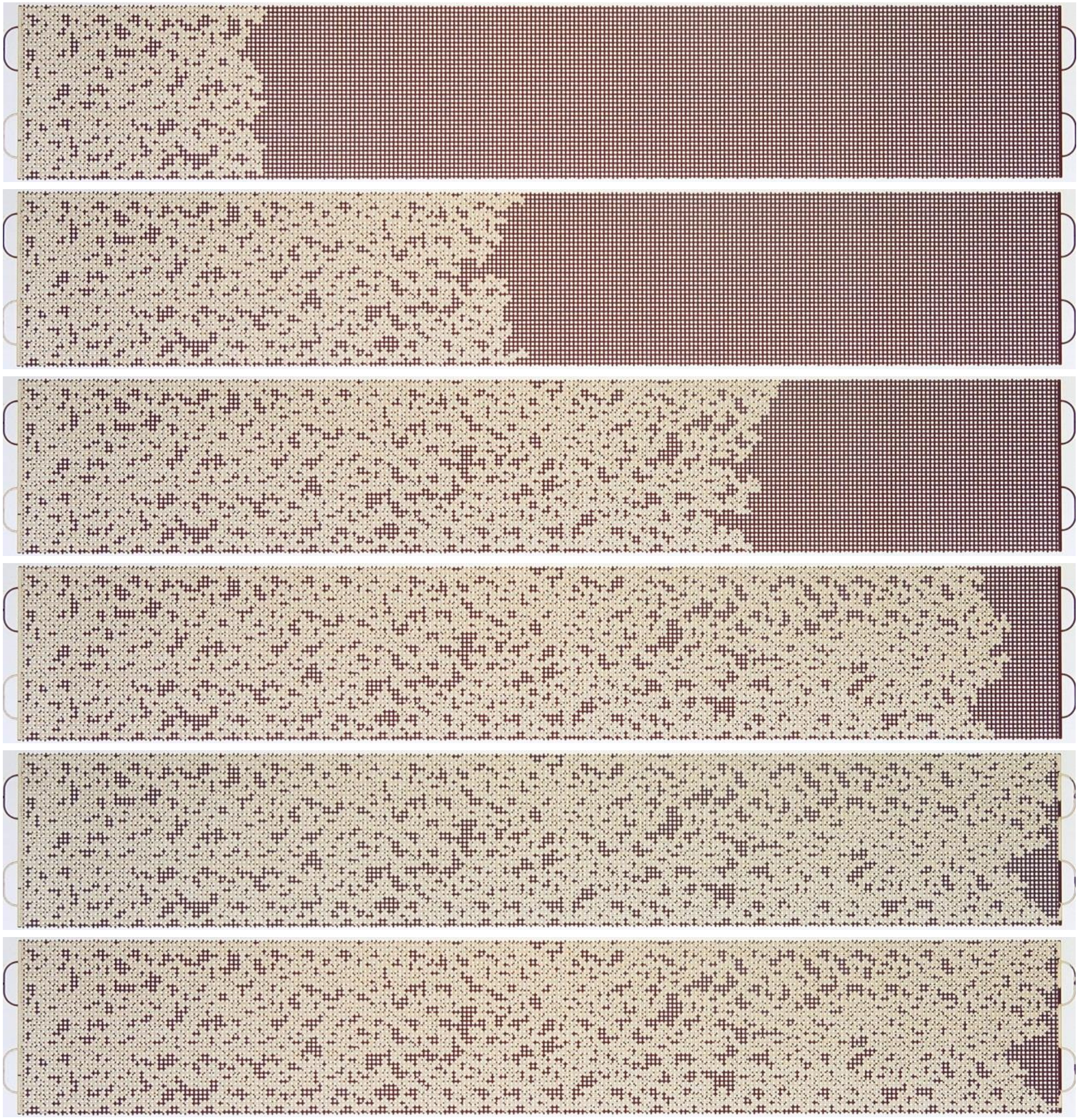

10 min

20 min

30 min

40 min

50 min

60 min

Figure S1. Photographs of the displacement process of an oil sample with a solution of polymer A2020 with a concentration of 0.1%.

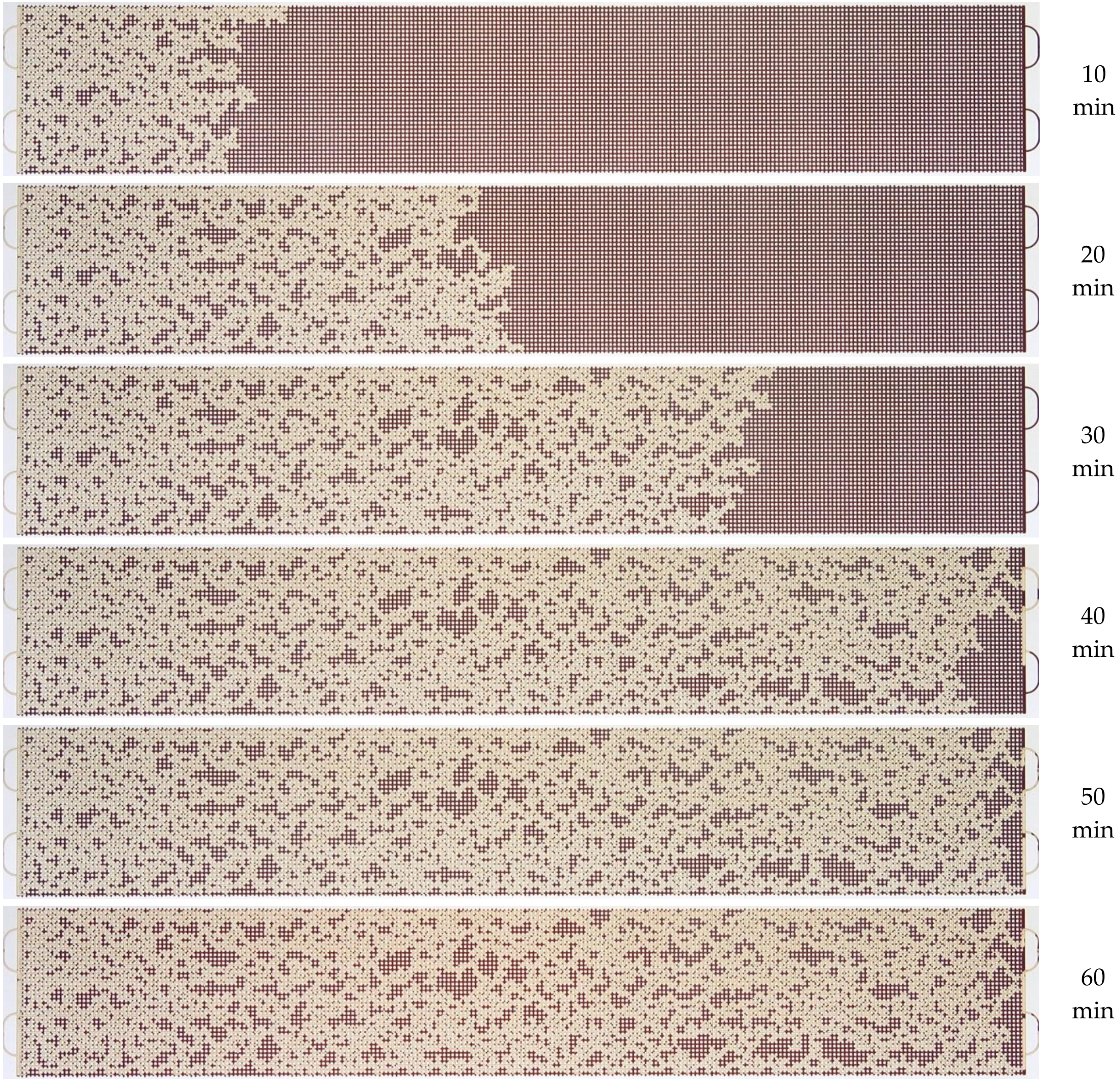

Figure S2. Photographs of the process of displacing an oil sample with a polymer 2515 solution with a concentration of 0.1%.

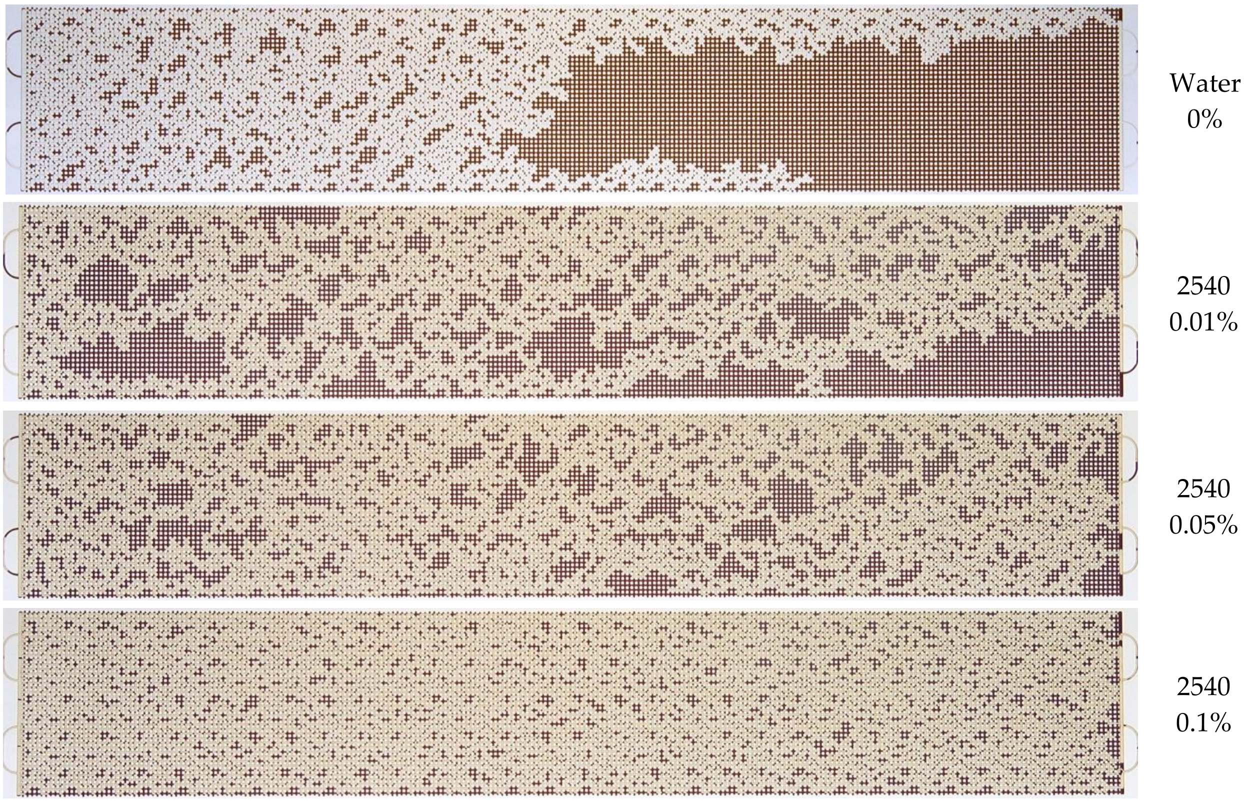

Figure S3. Photographs of the remaining oil distribution in the microfluidic chip after injection of a solution with different concentrations of polymer 2540.

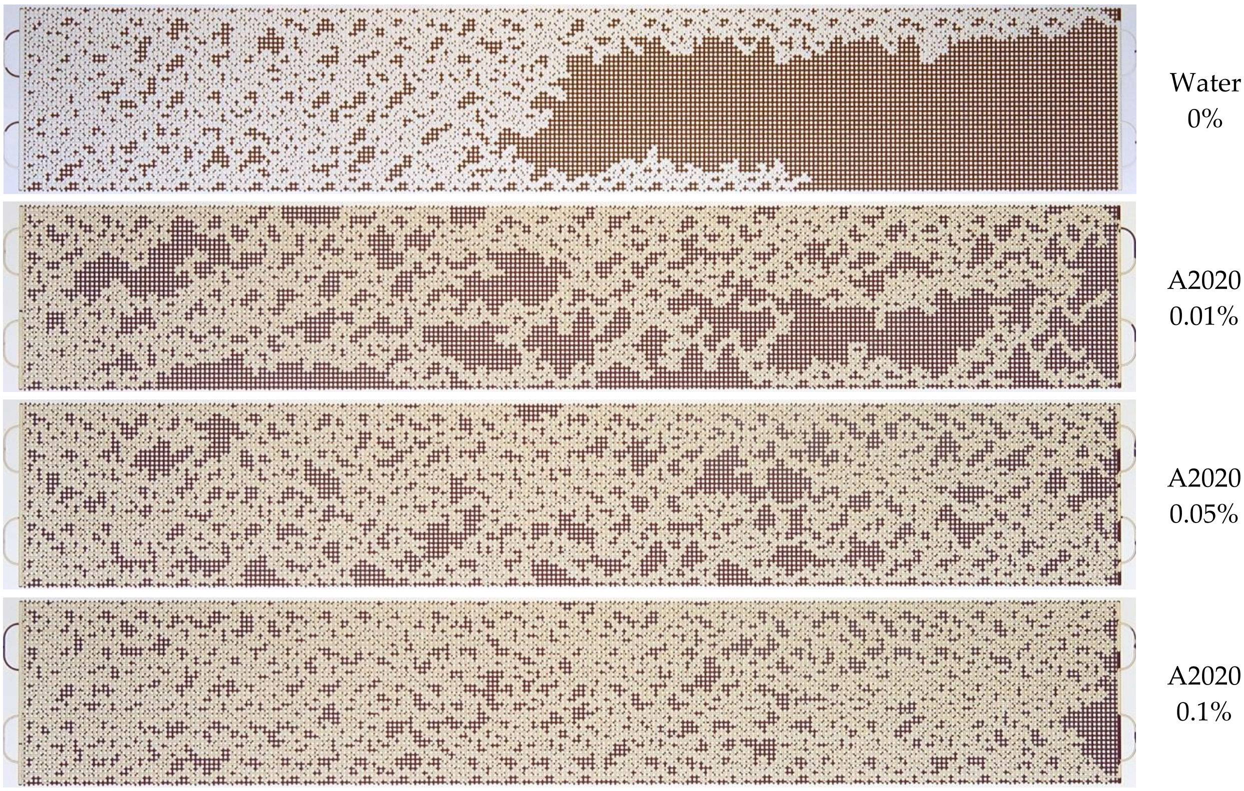

Figure S4. Photographs of the remaining oil distribution in the microfluidic chip after injection of a solution with different concentrations of polymer A2020.

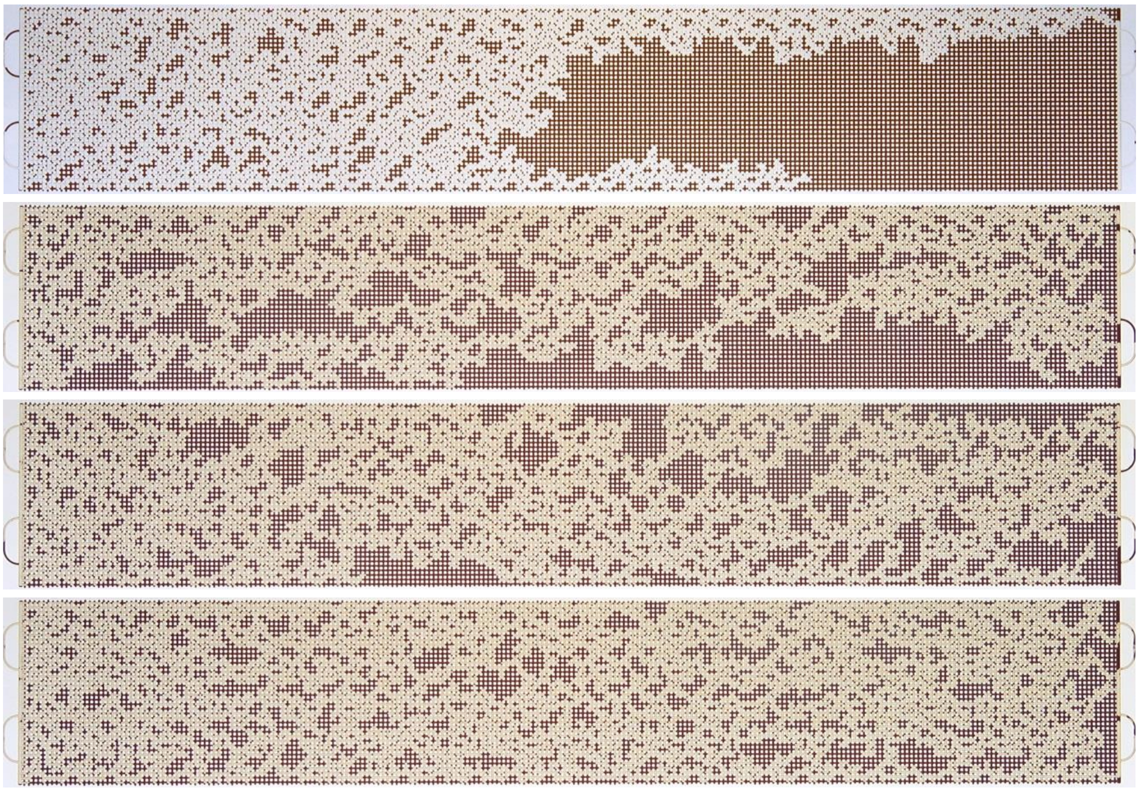

Water 0%

2515 0.01%

2515 0.05%

2515 0.1%

Figure S5. Photographs of the remaining oil distribution in the microfluidic chip after injection of a solution with different concentrations of polymer 2515.
